# OntoCAT -- simple ontology search and integration in Java, R and REST/JavaScript

**DOI:** 10.1186/1471-2105-12-218

**Published:** 2011-05-29

**Authors:** Tomasz Adamusiak, Tony Burdett, Natalja Kurbatova, K Joeri van der Velde, Niran Abeygunawardena, Despoina Antonakaki, Misha Kapushesky, Helen Parkinson, Morris A Swertz

**Affiliations:** 1 European Bioinformatics Institute, Wellcome Trust Genome Campus, Cambridge, CB10 1SD, UK; 2 Genomics Coordination Center, Department of Genetics, University Medical Center Groningen and Groningen Bioinformatics Center, University of Groningen, P.O. Box 30001, 9700 RB, Groningen, The Netherlands

## Abstract

**Background:**

Ontologies have become an essential asset in the bioinformatics toolbox and a number of ontology access resources are now available, for example, the EBI Ontology Lookup Service (OLS) and the NCBO BioPortal. However, these resources differ substantially in mode, ease of access, and ontology content. This makes it relatively difficult to access each ontology source separately, map their contents to research data, and much of this effort is being replicated across different research groups.

**Results:**

OntoCAT provides a seamless programming interface to query heterogeneous ontology resources including OLS and BioPortal, as well as user-specified local OWL and OBO files. Each resource is wrapped behind easy to learn Java, Bioconductor/R and REST web service commands enabling reuse and integration of ontology software efforts despite variation in technologies. It is also available as a stand-alone MOLGENIS database and a Google App Engine application.

**Conclusions:**

OntoCAT provides a robust, configurable solution for accessing ontology terms specified locally and from remote services, is available as a stand-alone tool and has been tested thoroughly in the ArrayExpress, MOLGENIS, EFO and Gen2Phen phenotype use cases.

**Availability:**

http://www.ontocat.org

## Background

Ontologies are increasingly used to annotate life science data [1], to improve search and integration [2], and to model complex biological knowledge unambiguously [3].

By definition, an ontology is a specification of a representational vocabulary for a shared domain of discourse - definitions of classes, relations, functions, and other objects [4] or in short it is an explicit specification of a conceptualisation [5]. For example, the Disease Ontology [6] and the NCI Thesaurus [7] are both examples of ontologies in the disease domain and *cancer *(accession: DOID:162) and *Malignant Neoplasm *(accession: C9305) are examples of equivalent concepts therein. When used this allows for unambiguous attribution of a particular experimental condition or sample characteristic.

In recent years the OBO Foundry community has succeeded in creating a valuable catalogue of orthogonal, cross-linked ontologies [8] for life sciences, of which the Gene Ontology [9] for integration of gene annotations is a well known example. However, there is still a considerable overlap especially in the areas of anatomy and disease, requiring much querying and integration when using ontologies in practice.

For the purpose of this article, ontology querying and integration is defined as integration of ontologies into software applications and the practical use of ontologies to annotate real data. This clarification is necessary as ontology integration can also be understood as the integration of ontologies when building new ontologies by reusing other ontologies, or integration of ontologies by merging different ontologies into a single one that unifies them [10].

Bioinformaticians integrating public domain ontologies into their work face the following issues: (1) there are no standardised identifying features that characterise ontologies from the user's perspective; (2) individual ontology resources do not follow the same logical organisation; (3) to search for appropriate ontologies requires expert knowledge [11,12]; and (4) programmatic ontology search is challenging. A WWW ontology broker has been proposed to solve some of the issues [11]. This was predated by an important effort in the biomedical domain, namely the Unified Medical Language System (UMLS), which encompasses a number of controlled vocabularies critical for the biomedical sciences and is still in active use for more than twenty years since its conception in 1986 [12].

**Figure 1:**
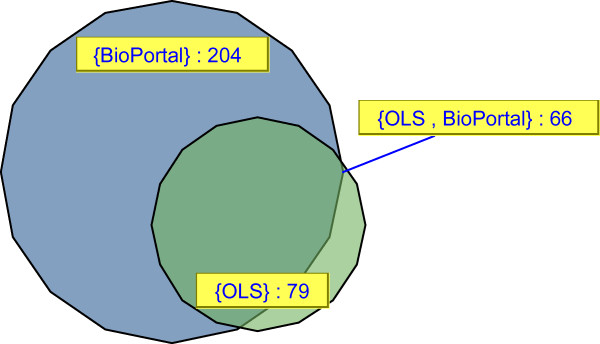
**Overlap between BioPortal and OLS**. Venn diagram representation showing overlap in content between BioPortal and EBI Ontology Lookup Service. The relative size of the circles relates to the number of ontologies stored in each repository (numbers shown in connected labels) and the overlap between the two repositories. 66 ontologies are shared between BioPortal and OLS [Created in VennMaster [16] on 27-07-2010]

There are now two major ontology repositories available: the EBI Ontology Lookup Service (OLS) [13,14] and the NCBO BioPortal [15]. Although there is some overlap in content between them (see Figure 1 [Created in VennMaster [16] on 27-07-2010]); some ontologies are only available in one repository so potentially both have to be queried to access all available ontologies. For example, Disease Ontology [6] is available in both repositories, but NCI Thesaurus [7] is not available in OLS, and Pride Controlled Vocabulary [17] does not exist in BioPortal. Table 1 highlights a case where both OLS and BioPortal serve the same ontology, but differ in features such as accessions, versions, or number of terms available. Moreover, many users also develop local ontologies for internal use, which are proprietary and cannot be published online, or are still in development and not suitable for public consumption. Those ontologies are often developed in OBO or OWL.

**Table 1:** Comparison of FMA ontology features available in BioPortal and OLS

	**OLS**	**BioPortal**
	
**Ontology name**	Foundational Model of Anatomy Ontology	Foundational Model of Anatomy
**Version**	0.1	3.0
**Release date**	15/09/2006	03/03/2009
**Ontology accession**	FMA	1053
**Format**	OBO	OWL
**Size in terms**	75149	81053
**Term accession**	FMAID (e.g. FMA:7088)	label (e.g. Heart)

The FMA ontology as shown here differs substantially in availability and structure between the two repositories [created on 27-07-2010].

It has been postulated that manual annotation is no longer sufficient to keep up with the current rate that new data are generated in the biomedical domain [18]. The field is undergoing rapid evolution, with the first generation of ontology development tools gaining wide adoption. Most notable examples in this area include Protégé [19], OBO-Edit [20], and OWL API [21]. Our experience is that building services to seamlessly access all these public repositories and local ontologies involves considerable effort, because these resources are still evolving or have rather advanced and heterogeneous programming structures. A new generation of tools geared more towards bioinformaticians is needed, with the focus on flattening the learning curve and making common tasks simpler.

Here we report the development of the software library for Common Ontology API Tasks (OntoCAT) that can be easily used from Java programs, Bioconductor/R [22] (*ontocat package*) and RESTful web services clients. Development as an open source community effort has helped to alleviate the challenges of accessing a fast evolving landscape of ontology resources and has already enabled large scale ontology integration in the Experimental Factor Ontology [23], ArrayExpress [24], MOLGENIS/XGAP [25] and Gen2Phen http://www.gen2phen.org use cases.

## Implementation

OntoCAT is an application suite that combines access to a large number of terms via a Java6 package, a REST interface, and an R interface, all available under the permissive LGPLv3 or Apache (R package) licenses for anyone to use and extend.

**Figure 2:**
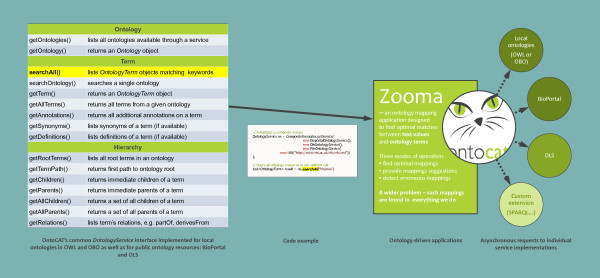
**Common workflow to integrate ontology resources**. The figure represents the common work flow when using OntoCAT's *OntologyService *interface (left-hand side) in ontology driven applications (right-hand side). OntoCAT is being used as a dispatcher to send off ontology related queries to several configurable resources, such as local ontology files, public repositories like OLS and BioPortal and is potentially easily extendable for other resources.

The OntoCAT Java package provides a generic *OntologyService *interface (described in detail in Figure 2) to query ontology sources. A code example is also provided in Figure 2. OntoCAT provides simple and easy-to-use API for BioPortal and OLS web services, and the OWL API [21] (*BioportalOntologyService*, *OlsOntologyService *and *FileOntologyService *respectively). Table 2 illustrates how the common interface maps to respective functionalities in supported resources. OBO ontologies are translated by OWL API into valid OWL by a dedicated OBO parser. This process results in a lossless version of the OBO ontology in OWL, as in terms of content OBO format can be considered a subset of OWL. In particular, synonyms and definitions are loaded into *synonym *and *def *OWL annotations respectively and are then available as synonyms and definitions in OntoCAT.

**Table 2:** Mappings between OntoCAT's interface and the underlying resources respective functionalities.

OntoCAT	OLS client	BioPortal REST	OWL API
	*QueryServiceLocator().getOntologyQuery() *	http://rest.bioontology.org/bioportal/	*OWLOntology *
	
		*ontology methods *	
**getOntologies() **	*.getOntologyNames()* Δ eager fetching with *.getOntology() *	./ontologies ?email=*email_address *	*.getOntologyID()* Δ
**getOntology() **	.*getOntologyNames()* *.getOntologyLoadDate()* *.getVersion() *	./virtual/ontology/*ontologyAccession *?email=*email_address *	*getOntologies().get(0) *

		*term methods *	

**searchAll() **	.*getPrefixedTermsByName(query, false*) Δ	./search/ ?query=*url-encoded_query* &sexactmatch=*[1/0]* &includeproperties*=[1/0]* &maxnumhits = 10000000 &email=*email_address*	.*getClassesInSignature()* *OWLClass.getAnnotations() *
**searchOntology() **	depending on the search options a combination of: 1) .*getTermsByExactName(query, ontolo-gyAccession)* 2) .*getTermsByName(query, ontologyAcces-sion, false)* 3) .*getTermsByAnnotationData(ontologyAccession, annotationType, query, fromDblValue, toDblValue), .getAnnotationStringValue()* Δ	./search/ ?query = url-encoded query &isexactmatch*=[1/0]* &includeproperties*=[1/0]* &maxnumhits = 10000000 &email=*email_address* &ontologyids=*ontologyAccession *	*searchAll()* there is only one ontology
**getTerm() **	Δ .*getTermById(termAccession, ontologyAccession) *	./virtual/ontology/*ontologyAccession* ?conceptid=*url-encoded termAccession* &email=*email_address *	*.getClassesInSignature()* Δ
		if *termAccession *was not found: ./search/?query=*url-encoded*_*termAccession* &isexactmatch = 1	
		&includeproperties = 1 &maxnumhits = 10000000	
		&email=*email_address *	
		&ontologyids=*ontologyAccession *	
**getAllTerms() **	no native support, slow *getRootTerms()* *getAllChildren() *	./virtual/ontology/*ontologyAccession*/all ?pagesize = 300 &pagenum=*pagenum* &email=*email_address *	.*getClassesInSignature()* Δ
**getAnnotations() **	.*getTermMetadata(termAccession, ontologyAccession) *	*getTerm() *	*OWLClass.getAnnotations() OWLAnnotation.getProperty().getIRI().toURI()* *OWLAnnotation.getValue().getLiteral() *
**getSynonyms() **	*getAnnotations()* Δ	*getTerm() * Δ	*getAnnotations()* Δ
**getDefinitions() **	*getAnnotations()* Δ	*getTerm() * Δ	*getAnnotations()* Δ

		*hierarchy methods *	

**getRootTerms() **	*.getRootTerms(ontologyAccession)* eager fetching with *getTerm() *	./virtual/ontology/*ontologyAccession* ?conceptid = root &email=*email_address* getChildren()	.*getClassesInSignature()* *OWLClass.getSuperClasses()* *getAnnotations() *
**getTermPath() **	non-recursive traversal of the path to root with *getParents()* Δ	./virtual/rootpath/*ontologyAccession*/url-encoded termAccession &email=*email_address* Δ	non-recursive traversal of the path to root with *getParents()* Δ
		eager fetching with *getTerm() *	
**getChildren() **	.*getTermChildren(termAccession, ontologyAccession, 1, null) *	*getTerm()* Δ	*OWLClass.getSubClasses() *
**getParents() **	*.getTermParents(termAccession, ontologyAccession) *	*getTerm()* Δ	*OWLClass.getSuperClasses() *
**getAllChildren() **		*getChildren() *called non-recursively	
**getAllParents() **		*getParents() *called non-recursively	
**getRelations() **	*.getTermXrefs(termAccession, ontology-Accession)*. *getTermRelations(termAccession, ontolo-gyAccession) *	not implemented in OntoCAT	not implemented in OntoCAT

Search defaults to non-exact matching and excludes properties, but this can be overridden by user with specific search options (see *SearchOptions *enum in the package). The Δ symbol signifies that some additional processing is performed on the original output of the underlying service to integrate the results with the OntoCAT's *OntologyService *interface. For example, in the case of OBO synonyms the appropriate property is found among the term's metadata and the additional context information is removed before the values are returned. Eager fetching means that when the underlying service returns only partial results, e.g. BioPortal's *rootpath *only provides term accessions, an additional query is issued to fully populate the result set. All hierarchy algorithms where noted are called in a non-recursive way to avoid memory issues for traversing large graphs in a recursive manner.

**Figure 3:**
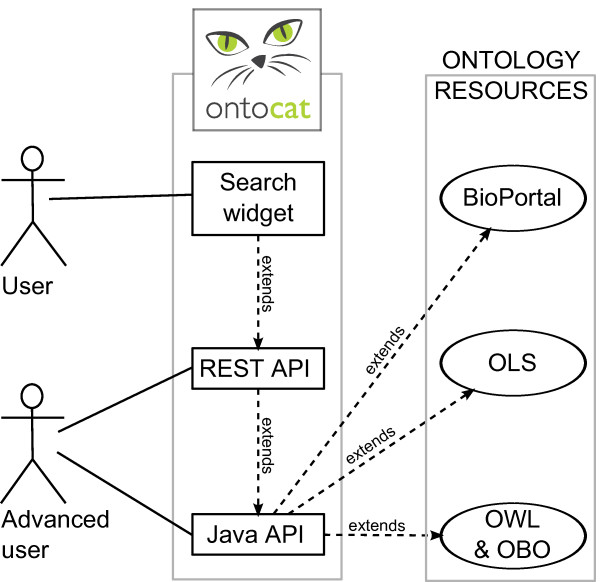
**Use case diagram**. Use case diagram of a simplified user interaction with existing ontology resources through OntoCAT.

Web applications can connect using REST or SOAP services as shown in Figure 3 and demonstrated in the online documentation via a simple html search widget. Researchers using R statistics can connect using the Bioconductor/R *ontocat *package [26]. Documentation is available at http://www.ontocat.org including programming examples and a complete javadoc. Simple demo applications showing OntoCAT for ontology searches with Google Web Toolkit on the Google App Engine framework and the MOLGENIS platform [27] are available at http://ontocat-web.appspot.com and http://www.ontocat.org/wiki/OntocatDownload. The Java API exposes the results as Java objects or Java primitives, the R package returns R objects, and the REST interface returns XML or JSON.

The basic *OntologyService *interface is further enhanced by a number of easy to use decorators (a design pattern that allows additional behaviour to be added to an existing object dynamically), which extend the primary functionality of the underlying resources to add caching, allow integrated searches across multiple ontology services or perform more specialised tasks like translation and sorting:

### CachedServiceDecorator

Adds two caching layers to every request relayed through the decorator. All requests test the first cache with a 24 hours expiry first. If no record is found the request is passed through to the original provider. If the original provider query fails the results are returned from the eternal cache (if available). This provides a fallback mechanism if an ontology resource becomes temporarily unavailable, e.g. due to maintenance downtime.

### CompositeDecorator

Allows searching across multiple ontology resources using a single *OntologyService *interface, effectively establishing another meta layer of abstraction. All underlying resources encompassing different repositories and local files are therefore available from a single access point. An example is provided in Figure 2 and in the online documentation. This service is multithreaded and requests are simultaneously sent off to all the underlying resources (the number of threads used is configurable). As in R the Java code cannot be multithreaded a dedicated implementation exists in *CompositeServiceNoThreads*.

### SortedSubsetDecorator

Adds sorting and subsetting capabilities to any search in the underlying service. This allows the prioritization and ranking of terms when searching over a specific selection of ontologies. The service is instantiated with a list of ontology accessions indicating suggested priority of use. All searches performed with the decorated *OntologyService *will be truncated to only include the results from this initial list of ontologies, and ranked in the original order the ontologies were specified.

### TranslatedOntologyService

Allows the mapping of term accessions from one local identifier namespace to a different one, e.g. between BioPortal and OLS schemas as shown in Table 1. This facilitates combining different ontologies under a single schema and resolves format inconsistencies, e.g. OWL NCBITaxon_1 versus OBO NCBITaxon:1. Behind the scenes an *OntologyIdTranslator *encapsulates a set of user defined *OntologyIdMappings*. Each mapping consists of a regular expression that uniquely identifies a term as belonging to a particular ontology, and another regular expression to translate the term accessions and ontology accessions into the required format.

Respective example code snippets are provided in the online documentation http://www.ontocat.org/wiki/OntocatGuide. Use of the decorator design pattern allows other ontology users to quickly extend OntoCAT, implementing additional functionality such as custom search filters.

## Results

OntoCAT has proven a valuable toolbox to search and integrate ontologies from heterogeneous sources on a large scale. Example use cases of OntoCAT for integrating ontologies with data include: harmonising and promoting consistency in data annotations, facilitating automated annotations, inferring additional information based on the knowledge conceptualisation, supporting complex user queries and user interfaces, nonsense detection and integrating external data. We report successful applications in the following real world scenarios:

### Use case: updating ontology properties

The Experimental Factor Ontology (EFO) [23] is an application focused ontology modelling the experimental factors in functional genomics experiments available from the EBI databases ArrayExpress [24] and the Gene Expression Atlas [28]. The development of EFO involves construction of mappings to multiple existing domain specific ontologies, such as the Disease Ontology [6] and Cell Type Ontology [29]. This is achieved using a combination of automated downloads of relevant ontology terms using OntoCAT. Periodically all the established cross-references in EFO to external ontologies have to be re-evaluated against BioPortal in order to detect changes in source ontologies in addition to importing new and extra annotations such as new synonyms and external definitions for EFO terms. Multithreading the OntoCAT requests allows our annotators to process and import extra information from over 20,000 external ontology terms in less than 10 minutes. Legacy OBO-like term accessions proved to be the biggest challenge, as they are not directly resolvable through BioPortal. For this reason custom implementations of the *TranslatedOntologyService *and *OntologyIdTranslator *classes were created saving great amounts of manual annotation and checking, and are available in the *uk.ac.ebi.efo.bioportal *package.

### Use case: annotating user supplied experimental values with ontology terms

The ArrayExpress Archive [24] and the Gene Expression Atlas [28] together contain over one million unique experiment annotations. These are all annotated with ontology terms from the EBI's pre-release version of the application ontology EFO, and where they do not exist in EFO they have to be checked against publicly available ontologies. This previously manual process has now been automated using the Zooma http://zooma.sf.net application, which extracts a list of unmapped terms from the source database and queries the local EFO pre-release OWL file and existing mapped data inside local databases. It returns perfect matches as automatic mappings, and also launches queries at BioPortal and OLS for unmapped terms. If several mappings to EFO are possible, these experiment annotations are flagged as requiring curation and periodically mapped by a member of the Atlas curation team. If no mappings to EFO were present, but possible mappings were acquired from other ontologies (using OntoCAT), then these are flagged as new suggestions and used in the development of EFO.

### Use case: local ontology management

OntoCAT has also been applied using the MOLGENIS software [27], and in particular on the eXtensible Genotype And Phenotype data platform, XGAP [25]. A user interface for ontology term search and management has been deployed as part of MOLGENIS code generation software and is used in XGAP and other models to allow users to search publically available ontologies and download terms to unambiguously annotate their experimental genotype, QTL or GWAS data http://www.xgap.org.

In addition a small html search widget can be used by bioinformaticians to add OntoCAT search and term selection anywhere in their web applications. Here the user is provided with a 'search' box where they can type in keywords and then a selection of matching candidate terms is loaded from OLS, BioPortal, OWL or OBO files for the user to choose from (exact results depend on how the underlying OntoCAT's REST service is configured). Working examples of both systems are provided in the online documentation http://www.ontocat.org/wiki/OntocatGuide.

### Use case: data analysis

Ontologies are used in data analysis as well as for annotation purposes and a new Bioconductor [22] package *ontocat *http://www.ontocat.org/wiki/r is now available to read in and query OWL/OBO format ontologies into R for use in downstream analyses. The *ontocat *package was released in Bioconductor 2.7 and comes with built-in offline support for EFO and also supports online BioPortal and OLS ontology queries.

## Discussion

### Simplicity and ease of use

OntoCAT was designed to make common use cases easy to implement while still enabling implementation of advanced algorithms. Many of such common tasks are demonstrated in code examples that are provided in the online documentation http://www.ontocat.org/wiki/OntocatGuide. OntoCAT was developed as a common access point for available resources and therefore does not incorporate all of their rich feature sets (see Table 3). New features are added to OntoCAT only when new use cases demand it and only for cases where the functionality in question is available across all the supported ontology resources or can be in some way emulated (see *Future work *below). The advantage of this approach is the *OntologyService *interface is more stable keeping the API very close to programmer's practice.

**Table 3:** Comparison of available features between existing ontology repositories and OntoCAT

	**OLS**	**BioPortal**	**OntoCAT**
	
**Web services**	SOAP	REST	REST
**Java API**	Yes		Yes
**Complexity (C)**	16	29	16
**Richness (R)**	177	1405	≥ 1582
**log R/C**	1.0	1.7	≥ 2.0
**OWL support**		Yes	Yes
**OBO support**	Yes	Yes	Yes
**Local ontologies**			Yes
**Open source**	Apache License	Apache License	LGPL v.3

Complexity is represented as a number of public methods available in the respective Java API interface or different web services signatures where the former is not available. Richness is measured as a number of hits returned from a non exact query for 'thymus'. A logarithm of richness and complexity quotient is provided as a single score to judge the relation between the two. It is higher the richer the source and less complex it is to access [created on 27-07-2010].

To further lower the barrier to practical use we followed the convention over configuration design approach. Whenever possible a default behaviour requiring zero configuration can be used by the developer minimizing the number of decisions that are needed to made, while not losing the flexibility of defining custom settings when necessary. This principle is strictly adhered throughout the package, but one notable example is the implementation of the Simple Knowledge Organization System (SKOS, http://www.w3.org/TR/skos-primer) properties. W3C recommends their usage to annotate definitions and synonyms in OWL, but few OWL ontologies adhere to this. OntoCAT fully supports SKOS properties. Whenever they are employed in an ontology; label, synonym and definition annotations will be recognised automatically. However, it is still possible to specify custom URI fragments on a per ontology basis when using a local file source.

Use of synonyms is another common task that has considerable significance in text mining activities [30]. The OBO standard for denoting synonyms and definitions is also fully supported without additional configuration. However, extra information available in OBO, in the form of cross-references to describe the origins of the synonym, synonym category and scope (EXACT, BROAD, NARROW, RELATED), are stripped from the results to be consistent with output from other services. The raw values can still be accessed through *getAnnotations() *if required.

Generally speaking ontologies consist of individuals (instances), classes (concepts), attributes, and relations. In order to avoid the confusion in the implementation - the word "concept" is avoided altogether in the implementation, and "term" is used instead to denote both individuals and concepts. Here an *ontology term *is defined as a tuple consisting of (1) term accession, (2) term label, and (3) ontology accession that the particular term belongs to. Ideally a term accession should be enough to uniquely identify a term thereby rendering term label and ontology accession redundant. However, this is not the case when label is the accession as shown for the FMA ontology in BioPortal (see Table 1). Storing an ontology accession helps to resolve the actual ontology the term belongs to. The label is only recorded for convenience as in most cases it is used for text mining, query support or indexing. In fact, in this case the label forms the fragment of the term URI, but the term cannot be retrieved solely by its URI as the REST query additionally requires the Bioportal-assigned ontology accession.

In theory a URI should be enough to uniquely identify an ontology term across all services solving the complex issue of integrating data annotated using different ontology resources and in the semantic web applications; wide adoption would render the *TranslatedOntologyService *unnecessary. In BioPortal it is already possible to use URIs (called *full IDs*) in place of the short term accessions, however at present terms cannot be retrieved solely by their URIs as the REST interface requires the BioPortal-assigned ontology accession to be provided additionally. While this is a minor issue that is likely to be resolved in the future, there are a number of other factors preventing wide adoption of resource identifiers. Although OWL ontologies fully support term URIs already, OBO format provides only rudimentary support. OBO community is in the process of embracing Persistent Uniform Resource Locators (PURLs) to encode namespaces and this issue is likely to be mitigated in the future. However, while all PURLs are URIs, not all URIs are PURLs, in particular URIs are not limited to http protocol. To add to the confusion OWL 2 specification replaced URIs with Internationalized Resource Identifier (IRIs), which potentially are not backward compatible with URIs (Unicode vs. ASCII encodings). Ideally, a consensus is also required in whether and how to report the ontology version within the URI and there is also the issue of URI stability between versions, e.g.:

• *FMA 3.1 *- http://sig.uw.edu/fma#Anatomical_entity

• *FMA 3.0 *- http://sig.biostr.washington.edu/fma3.0#Anatomical_entity

• *FMA 1.4 *- http://bioontology.org/projects/ontologies/fma/fma20OwlDlComponent#Anatomical_entity

### Common functionality and integration

No term-level versioning information is stored at present as we have no use cases where this would be particularly useful. This information is only available through BioPortal, no such feature exists in OLS, and there are no standards to code against to identify versions in OWL. A common practice in the community is that any substantial change to a term is recorded by assigning a new term accession and obsoleting the original one. It is not strictly defined what makes a change substantial, e.g. debatable whether changing a class' position in ontology hierarchy warrants a new accession. Alternatively, term annotations could be used as a proxy for detecting changes. A version clash could be detected where a single annotation (e.g. label or synonym) changes between two retrievals of the same ontology term. BioPortal exclusively provides access to previous versions of ontologies, however their feature set is limited compared to the most recent versions (accessed by the so called *virtual ontology id*), i.e., hierarchy and search services are not available. For example, for ontologies following a daily release cycle it is impractical to reindex them so often. However, since BioPortal stores all the ontologies that were ever processed in a downloadable format, it is possible to use this facility by passing any of the version links provided in the BioPortal's ontology summary view into the OntoCAT's *FileOntologyService*. This allows working with a specific version of the ontology, not necessarily the latest one, including hierarchy and search functionalities. It is particularly useful in ontology development process for regression testing. Example 14 in the online documentation shows how two different versions of EFO can be compared in such a way. Replicas of ontologies, i.e., the same ontology present in both repositories, are treated as different ontologies as they have different ontology accessions. BioPortal assigns unique numeric identifiers, whereas OLS uses the abbreviation of the ontology name. These ontology accessions cannot be resolved against each other automatically and in practice this should not be attempted due to differences shown in Table 1. In fact, not a single ontology between BioPortal and OLS shares the same ontology accession thus a manual mapping is required. Example 7 in the online documentation demonstrates how one ontology namespace could be translated into another using the aforementioned *TranslatedOntologyService *and the two replicas integrated. When searching across both the repositories combined it is also possible to ignore one of the copies selectively with the *SortedSubsetDecorator *(see example 4 in the online documentation). Due to significant differences in the same ontology between the sources as demonstrated in Table 1 integrating the results is not recommended in practice. Nevertheless, replication is handled gracefully without additional overhead because of the unique ontology accessions. That is if a user tries to access the term GO:0043227 given its term (GO:0043227) and ontology (GO) accessions, only a single version specific to OLS (as identified by the ontology accession) will be returned.

The following features were implemented directly in OntoCAT as the underlying ontology resources did not support them, and we had use cases for these.

• Neither the OLS client nor OWL API provide *getSynonyms(), getDefinitions ()*, *getAllParents(), getAllChildren()*, and *getAllTerms() *methods natively; these were implemented using available meta data. The functionality of searching annotations across the whole repository does not exist in OLS either and cannot easily be emulated. A warning is issued whenever a user attempts this.

• The OWL API does not support search methods natively. These were implemented to provide the full functionality of searching properties and non exact matching. For example, a search for 'thymus' with search options: non exact, not including properties involves iterating through all the classes' labels in an ontology to verify which label contains the specified keyword. A class label is defined in this context in order of preference and availability as a user-specified property, rdfs:label, skos:prefLabel, or a fragment of the class URI.

Only in-memory caching of ontology terms is performed, and the whole cache is searched when a query is issued. We have not observed scalability issues on the local level when using ontologies up to 40000 terms that would warrant building an index to aid the queries. However, users could potentially employ OntoCAT as a means of populating an external index, e.g. Lucene-based http://lucene.apache.org, which we also did to enable query expansion in our MOLGENIS database. The in-memory cache is used to access OWL classes by their accessions as a convenience method for bypassing the OWL API requirement for fetching a class by its full URI. It is in theory potentially unsafe and could lead to collisions, i.e., OWL format allows for two classes to share the accession providing they are in different namespaces and in such a case one of the terms would be discarded. In practice this is unlikely to happen as most ontologies use globally unique term identifiers and this shortcut makes the interface easier to work with and to understand for consumers of OBO ontologies, where the usage of URIs is not as widespread.

A disadvantage of using web services over local access is that they are slower. It could be argued that providing an extra layer of abstraction would slow them down even further. However, in our tests OntoCAT only adds on average 9% overhead to a BioPortal query and less than 1% to an OLS query when compared to using the underlying source APIs directly. An OntoCAT term search takes on average 240 ms in BioPortal and 340 ms in OLS. These timings include the additional overhead OntoCAT imposes on the repository services 10 ms ± 5 ms for BioPortal and less than 1 ms for OLS. Moreover, with OntoCAT the queries can be easily parallelised, intermediate results cached or even whole ontologies downloaded from BioPortal for local querying, which results in orders of magnitude efficiency improvements that eclipse these minimal adverse overhead issues. Furthermore, use of both web services provides some redundancy in case of service dropout.

To generate these results a list of 100 random 13 character long alphanumeric strings was created. Random strings were used in order to prevent the repository from returning a previously cached result and to bypass the internal caching. These were then all used as a search keyword to query BioPortal and OLS. The standard high precision system timer was used to time each query and all the results were then averaged and corrected for average roundtrip time (measured by pinging the respective servers) to give the final figures. Complete code is available in the *ServiceProfiler *class. Please note that this cannot be considered a valid comparison of responsiveness of the two repositories, as the tests have not been performed across all the services and over extended period of time, but rather it is given here as a means of estimating expected OntoCAT's performance.

OntoCAT can handle arbitrary large ontologies, as long as OWL API has enough dedicated heap space available to parse them. For example, FMA (81053 terms) can be loaded with as little as 2GB of memory dedicated to the Java Virtual Machine (JVM), which is not unreasonable to expect from a modern laptop, and even less so on an academic cluster. If there is not enough memory available OntoCAT internally will catch the *OutOfMemoryError *exception on ontology loading and will inform the user which JVM settings have to be adjusted.

### Future work

The current repertoire of supported ontology resources could easily be extended when new resources become available and appropriate use cases are identified. Such services would only need to implement the *OntologyService *interface to immediately become aligned with pre-existing resources and allow for their seamless interchangeability. For example, we envision the creation of a dedicated SPARQL wrapper to allow for integration with RDF triplestores.

It is also possible to extend the functionality of an individual resource separately without impeding the core functionality. Example 8 in the online documentation demonstrates searching of a sub-tree of an ontology, which is possible using the *BioportalOntologyService *object directly, but was not promoted to the main *OntologyService *interface, as this functionality remains specific to BioPortal. This particular feature was implemented when requested by a user, and further extensions could be made in a similar fashion.

## Conclusions

OntoCAT is a comprehensive software package that allows bioinformaticians to uniformly access ontology terms from diverse public repositories and private file sources using simple Java, R and REST web service commands. OntoCAT is used in a growing list of applications including the Experimental Factor Ontology [23] development, the eXtensible Genotype And Phenotype data platform, XGAP [25], the Zooma annotation tool http://zooma.sf.net and the ArrayExpress [24] and Gene Expression Atlas [28] annotation systems.

## Availability and requirements

**Project name: **OntoCAT

**Home page: **http://www.ontocat.org

**Operating system: **Platform independent

**Programming language: **Java6

**License: **LGPLv3

## Authors' contributions

TA and MS both contributed equally to the conception and design of OntoCAT and this manuscript. HP participated in the design of OntoCAT and helped to draft the manuscript. TB provided code review and some of the requirements. NK contributed to the development of the OntoCAT Java API. NK and MK developed the *ontocat *Bioconductor/R package. DA developed the REST interface and html search widget. JV created the ontology manager and browser extension for MOLGENIS database. NA conceived the ranking and caching layer and participated in its implementation. TB, NK, JV, NA, DA and MK provided testing of OntoCAT and contributed to the manuscript. All authors read and approved the final manuscript.
